# Interaction between CYP1A1/CYP17A1 polymorphisms and parental risk factors in the risk of hypospadias in a Chinese population

**DOI:** 10.1038/s41598-019-40755-8

**Published:** 2019-03-11

**Authors:** Yaping Mao, Kang Zhang, Lin Ma, Xiaoyun Yun, Fengrong Ou, Ge Liu, Yi Yang, Yumin Zhang, Xiucong Pei, Zhiwen Duan, Mingyue Ma

**Affiliations:** 1 Department of Toxicology, School of Public Heath, Shenyang Medical College, Shenyang, Liaoning Province 110034 China; 2 Editorial Department of Journal of Shenyang Medical College, Shenyang Medical College, Shenyang, Liaoning Province 110034 China; 3 Department of Clinical Nutrition, The First Affiliated Hospital of China Medical University, Shenyang, Liaoning Province 110001 China; 4 Department of Pediatric Surgery, Shengjing Hospital of China Medical University, Shenyang, Liaoning Province 110004 China

## Abstract

Hypospadias (HS) is a common congenital malformation of the genitourinary tract in males and its etiology is viewed as multifactorial, and studies about gene-environment interaction in the etiology of HS are rare. A total of 152 cases and 151 controls were selected in the present study. Information before and during pregnancy from questionnaires finished by mothers of subjects were extracted, and the relating data were analyzed to determine the risk factors of HS. Meanwhile, maternal genomic DNA was genotyped for the single nucleotide polymorphisms (SNPs) of *CYP1A1* rs1048943 and *CYP17A1* rs4919686. Results of multivariable logistic regression analyses showed that several factors were associated with hypospadias risk. Analysis of the distributions of SNPs in *CYP1A1* and *CYP17A1* genes showed that the mutant genotype CC (OR = 4.87) of CYP1A1 rs1048943, and mutant genotype CC (OR = 5.82), recessive genotype AC + CC (OR = 2.17) and allele C (OR = 1.77) of CYP17A1 rs4919686 significantly increased the risk of HS. In addition, the additive gene-environment interactions were also found in several models. Several maternal risk factors that are associated with HS risk can interact with *CYP1A1/CYP17A1* polymorphisms, which lead to infants vulnerable to occurrence of HS in Chinese populations.

## Introduction

Hypospadias (HS), one of the most common congenital reproductive malformations, is a urethral opening on the ventral surface of the penis or on the scrotum or the perineum due to incomplete fusion of the urethral folds^1^. With the seriousness of environmental pollution intensifying in different countries, a number of related-studies have reported an increasing trend in prevalence of HS over time^2–5^. While in some sites, Europe^6^ and Australia^7^, incidence rates of HS was stable comparatively. For China, the overall prevalence of HS is 7.64 per 10 000 infants, and the eastern region was higher than the western^5^.

The etiology of HS has proven to be multifactorial, involving genetic, endocrine and environmental factors^8,9^. Furthermore, Marrocco *et al*. showed that the interaction between genotype and environmental factors mainly increased the risk of HS^10^, and it might not be induced by genetic or environmental single factors. However, most studies only focused on a single type of risk factor for HS, environmental risk factor such as consumed alcohol, drugs intake during pregnancy, proximity to agricultural pesticide, maternal age (>35), and hypertension during pregnancy, or genetic risk factors such as single nucleotide polymorphism in genes related to metabolism of endogenous estrogens and normal urethral development^11–15^. The importance of gene-environment interaction in the etiology of HS was only investigated in few studies, and the related genes include steroid 5 alpha-reductase (*SRD5A2*), activating transcription factor 3 (*ATF3*) and methylenetetrahydrofolate reductase (*MTHFR*)^16,17^. One possible explanation for this disease is environmental contamination. As many pollutants are metabolized by detoxifying enzymes, also polymorphisms of their encoding genes, which are able to interfere with their catalytic efficiency, may play a noteworthy role in the urogenital tissue formation^10^. The cytochrome P450 (CYP) enzymes can metabolize various exogenous substances such as environmental chemicals and pollutants, and two enzymes of this superfamily, the aryl hydrocarbon hydroxylase (CYP1A1) and the bifunctional 17α-hydroxylase/17, 20-lyase (CYP17A1), play major roles in this metabolism^18,19^.

*CYP1A1* is located on chromosome 15q22-q24, and is a well-studied phase I enzyme^18^. CYP1A1 can metabolically transform not only environmental chemicals but also endogenous estrogens into more hydrophilic compounds^20,21^. The T/C mutation in exon 7 of *CYP1A1* (rs1048943), which is induced by some environmental chemicals such as TCDD (2,3,7,8-tetrachlorodibenzo-p-dioxin) and benzo[a]pyrene (BaP), leads to an amino acid substitution from isoleucine (Ile) to valine (Val) at codon 462^16,22,23^. CYP1A1 I462V polymorphism shows the effect of enhancing catalytic activity^24^. Nebert *et al*. reported that CYP1A1 was involved in the clearance and bioactivation of endogenous steroids including estradiol, progesterone and pregnenolone, which are necessary for the normal development of genital gland^22^. Because normal urethral development depends on the delicate balance of these endogenous hormones, metabolites of estrogens or other hormones formed by CYP1A1 might play roles in the development of HS^25^.

CYP17A1 is another key enzyme in steroid hormone biosynthesis pathway^26^. The enzyme has both 17α-hydroxylase activity and 17, 20-lyase activity^20^. *CYP17A1* gene is located on chromosome 10q24.3, and is mostly expressed in adrenals and gonads, as well as plays an important role in normal physiological events such as adrenarche and puberty^27–29^ CYP17A1 is required for androgen and oestrogen synthesis^30,31^. Given that androgens serve as precursors to estrogens, normal estrogen signaling is also dependent on CYP17A1, which suggested that it could regulate the normal development of urethra of fetus^32^. Mutations in CYP17A1 can result in the loss of both 17-hydroxylase and 17,20-lyase activities, and then the loss of all or most CYP17A1 activities leads to the deficiency of glucocorticoid cortisol and androgens (and in turn estrogens), which might play roles in the development of HS^15^.

And so far, the interaction of *CYP1A1/CYP17A1*-environment in the risk of HS has not been reported in Chinese population, and we hypothesized that *CYP1A1*/*CYP17A1*-environment interaction could increase the risk of HS by changing the delicate balance of hormones. So we performed a hospital-based case-control study in Asian population of China to investigate the role of the two genes’ polymorphisms and their interaction with environmental risk factors before and during pregnancy in the etiology of HS.

## Materials and Methods

### Study Population

Cases (152) were live born children diagnosed with HS at the Department of Pediatric Urology of Shengjing Hospital of China Medical University between August 2016 and September 2017. Controls (151) were randomly selected from the same hospital during the same period. Oral epithelial cells or saliva were collected from mothers of the subjects. Mothers were also invited to fulfill questionnaires concerning demographics, family history, health and lifestyle before and during pregnancy.

Case selection criteria were with isolated HS according to the International Classification of Diseases (ICD)-11^33^. The results about whether newborns had any congenital malformations were got by obstetricians and the congenital reproductive malformations were further diagnosed by urologists. All participants were native Chinese. Cases and controls with a syndrome or chromosome abnormality and cases with a known cause of hypospadias were excluded. The specific exclusion criteria were true hermaphroditism, chordee alone, and adrenogenital syndrome. Meanwhile, to ensure independent analyses, we selected only one case or control per-family in the study after excluding the youngest brother or one of the twin brothers randomly.

### DNA extraction and genotyping

We collected the oral epithelial cells or saliva from each subject’s mother by using throat swab to scrub two side of oral cavity after gargling with rinsing twice. Genomic DNA was extracted by using EZ1 DNA Investigator Kit (QIAGEN, Germany) and was stored at −80 °C immediately for further genotype analysis.

DNA samples were genotyped for the single nucleotide polymorphisms (SNPs) of *CYP1A1* rs1048943 (MAF = 0.13) and *CYP17A1* rs4919686 (MAF = 0.11) using the 5′-nuclease SNP Genotyping Assay (assay ID: C_25624888_50 and C_11201596_10, Applied Biosystems, Foster City, CA). The TaqMan SNP genotyping assays were performed by Applied Biosystems 7500 Fast Real-Time PCR System, which were carried out in 96-well plates in a 5 μl reaction volume containing 10 ng genomic DNA, 0.125 μl assay mix (20×), 2.5 μl Taqman universal PCR mastermix (2×), and 1.375 μl milli-Q. Cycling conditions were consisted of a pre-PCR read at 60 °C for 60 sec, then pre-denaturation step at 95 °C for 10 min, followed by 40 cycles of denaturation at 95 °C for 15 sec and annealing and extension at 60 °C for 60 sec, ended by post-PCR read at 60 °C for 60 sec. FAM (480/520 nm) and VIC (530/560 nm) fluorescence levels of PCR products were measured at 60 °C for 1 min. Genotyping was carried out in the Key Laboratory of Environmental Pollution and Microecology of Liaoning Province for quality control. After PCR, samples were genotyped using allelic discrimination in an Applied Biosystems 7500 Fast Real-Time PCR System.

### Questionnaire data

After signing informed consent, parents were interviewed one-to-one with the predesigned questionnaires by interviewer who was pre-trained by graduate advisor on the content of the questionnaire. Questionnaires contained a variety of questions about lifestyle, environmental exposure and health status before and during pregnancy, and these information were analyzed to determine the individual and environmental risk factors for HS. Infant characteristics included gender, year of birth, birth weight, premature delivery or not. Parental characteristics were family residence when mother got pregnant (rural/urban), educational level (junior high school education, senior high school education, undergraduate education, postgraduate education), history of smoking and drinking. Besides, maternal characteristics also included age at delivery (<25, 25–29, 30–34, or ≥35 years old), drug intake before and during pregnancy (such as antibiotics, anti-abortion drugs, antiepileptic drugs, contraceptive drugs, hypnotics, anti-constipation drugs, and cough and cold medications), progesterone intake, pesticides exposure, pre-pregnancy body mass index (BMI) (<18.5, 18.5–24.9, 25.0–29.9, and ≥30.0 kg/m^2^), irregular menstruation (during 12 months before pregnancy), occurrence of abnormalities during pregnancy (gestational diabetes, amniotic fluid anomaly, toxemia of pregnancy, premature labor, influenza and others), diseases during pregnancy (gynecological disease, bronchial asthma, diabetes, hypertension, liver disease, heart disease, cancer, thyroid disease, epilepsy, and others).

### Statistical analysis

The *CYP1A1* rs1048943 and *CYP17A1* rs4919686 genotypes frequencies in controls were tested by Hardy-Weinberg equilibrium (HWE), and *P* < 0.05 was regarded as a significant deviation from HWE^34^. The crude and adjusted odds ratios (ORs) with 95% confidence intervals (95% CIs) for the independent associations of HS with parental risk factors before and during pregnancy and maternal *CYP1A1* rs1048943/*CYP17A1* rs4919686 genotypes were calculated by univariable and multivariable logistic regression analyses. Meanwhile, the role of interactions between maternal risk factors and *CYP1A1* rs1048943/*CYP17A1* rs4919686 polymorphisms in the etiology of HS were examined as well. Relative excess risk of interaction (RERI) and attributable proportion due to interaction (AP) were calculated to assess interaction on an additive scale using the method proposed by Rothman and Excel spreadsheet developed by Andersson *et al*.^35,36^.

In order to get precise results of the present study, we considered maternal age at delivery, maternal residence during pregnancy as potential confounding factors in this analysis. And the risk estimation was adjusted by these confounding factors in both univariable and multivariable logistic regression analyses. SPSS 22.0 software for Windows (IBM SPSS, Chicago, IL, USA) was used to perform statistical analyses.

### Statement of methods

Our study was approved by the Ethics Committee of Shenyang Medical College and written informed consent was obtained from each participant. All researches were performed in accordance with relevant guidelines/regulations.

## Results

### Study population

The participation rates were 85% for cases and 75% for controls, resulting in a total of 163 cases and 159 controls that were available for this study. To ensure the accuracy of the investigation, we excluded 11 cases for chromosome abnormality or a known cause of HS and 8 controls for disease (such as cryptorchidism) might induced by the same cause of HS. After the exclusion step, 152 cases and 151 controls were available for risk factors analyses, while because of the failed DNA extraction of several subjects (<10%), only 148 DNA samples of cases and 147 DNA samples of controls were obtained to perform SNPs genotyping. As shown in Table 1, compared with the mothers of controls, mothers of cases were older at time of delivery (28.22 vs 27.54), had a higher proportion of living in rural areas (36.2% vs 29.8%), had a lower educational level (junior and senior high: 61.8% vs 42.4%), and also had a higher proportion of abnormal BMI (28.1% vs 21.2%).

**Table 1:** Demographic characteristics for case and control groups.

Characteristics	Cases (N = 152)%	Controls (N = 151)%
**Infant characteristics**		
Year of birth		
<2008	31 (20.4%)	30 (19.9%)
2008–2010	54 (35.5%)	42 (27.8%)
2011–2013	32 (21.1%)	64 (42.4%)
>2013	35 (23.0%)	15 (9.9%)
**Maternal characteristics**		
Age at delivery		
<25 years	36 (23.7%)	38 (25.2%)
25–29 years	57 (37.5%)	59 (39.1%)
30–34 years	42 (27.6%)	45 (29.8%)
≥35 years	17 (11.2%)	9 (6.0%)
Residence		
Rural	55 (36.2%)	45 (29.8%)
Urban	97 (63.8%)	106 (70.2%)
Educational level		
Junior high	36 (23.7%)	26 (17.2%)
Senior high	58 (38.1%)	38 (25.2%)
Undergraduate	38 (25.0%)	47 (31.1%)
Postgraduate	20 (13.2%)	40 (26.5%)
Pre-pregnancy BMI		
Underweight (<18.5 kg/m^2^)	32 (21.1%)	16 (10.6%)
Normal (18.5–24.9 kg/m^2^)	99 (71.9%)	119 (78.8%)
Overweight (25.0–29.9 kg/m^2^)	17 (11.2%)	15 (9.9%)
Obese (≥30 kg/m^2^)	4 (2.6%)	1 (0.7%)

### Association of parental factors with hypospadias

The results of univariable logistic regression analyses showed that there were many significant differences between cases and controls, as showed in Table 2. Infants with low birth weight (LBW) (OR = 6.40) and premature delivery (OR = 3.58) were associated with the increased risk of HS. Parents with higher education could decrease the risk of HS (fathers with undergraduate level: OR = 0.39, 95%CI: 0.20–0.78, *P* = 0.01; fathers with postgraduate level: OR = 0.36, 95%CI: 0.17–0.76, *P* = 0.01; mothers with postgraduate level: OR = 0.36, 95%CI: 0.17–0.79, *P* = 0.01). Mothers with irregular menstruation, drug intake (including antibiotics, contraceptive drugs, hypnotics, anti-constipation drugs, and cold medications), cigarette smoking (≥1 cigarettes/day), occupational passive smoking exposure and underweight BMI (<18.5 kg/m^2^) before conception contributed to a higher risk of HS in offspring. Similarly, maternal factors during pregnancy such as illness (including gynecological disease, bronchial asthma, urethritis, anemia, diabetes, hypertension and liver disease), occurrence of abnormalities, progesterone intake, and occupational passive smoking exposure were also associated with the increased risk of HS. The results also showed that household monthly income was associated with HS.

**Table 2:** Univariable logistic regression analyses of potential risk factors for HS.

Risk Factors		Cases N (%)	Controls N (%)	OR (95%CI)	P*
**Of Infant**					
Low Birth Weight	No	102 (67.1%)	140 (92.7%)	1.00 (referent)	
	Yes	50 (32.9%)	11 (7.3%)	6.40 (1.90–6.74)	**0.00**
Premature delivery	No	104 (69.3%)	135 (89.4%)		
	Yes	46 (30.7%)	16 (10.6%)	3.58 (1.90–6.74)	**0.00**
**Of Father**					
Educational level	Junior High	34 (22.4%)	20 (13.2%)	1.00 (referent)	
	Senior High	55 (36.2%)	35 (23.2%)	0.98 (0.49–1.98)	0.96
	Undergraduate	40 (26.3%)	61 (40.4%)	0.39 (0.20–0.78)	**0.01**
	Postgraduate	23 (15.1%)	35 (23.2%)	0.36 (0.17–0.76)	**0.01**
**Of Mother**					
Irregular menstruation BP^a^	No	116 (73.6%)	138 (91.4%)	1.00 (referent)	
	Yes	36 (23.7%)	13 (8.6%)	3.26 (1.63–6.51)	**0.001**
Drug intake BP^b^	No	113 (74.3%)	127 (84.1%)	1.00 (referent)	
	Yes	39 (25.7%)	24 (15.9%)	1.76 (0.99–3.15)	**0.05**
Occurrence of illness DP^c^	No	114 (75%)	131 (86.8%)	1.00 (referent)	
	Yes	38 (25%)	20 (13.2%)	2.01 (1.10–3.71)	**0.02**
Occurrence of abnormalities DP	No	97 (63.8%)	123 (81.5%)	1.00 (referent)	
	Yes	55 (36.2%)	28 (18.5%)	2.47 (1.44–4.22)	**0.001**
Progesterone intake DP	No	112 (73.7%)	128 (73.7%)	1.00 (referent)	
	Yes	40 (26.3%)	23 (15.2%)	1.89 (1.05–3.39)	**0.03**
Cigarette smoking BP	No	143 (94.1%)	150 (99.3%)	1.00 (referent)	
	Yes	9 (5.9%)	1 (0.7%)	8.77 (1.09–70.53)	**0.04**
Passive smoking exposure BP^d^	No	96 (63.2%)	95 (62.9%)	1.00 (referent)	
	Seldom	34 (22.4%)	47 (31.1%)	0.74 (0.43–1.27)	0.28
	Frequently	22 (14.5%)	9 (6.0%)	2.58 (1.11–6.00)	**0.03**
Passive smoking exposure DP^d^	No	112 (73.7%)	99 (65.6%)	1.00 (referent)	
	Seldom	25 (16.4%)	44 (29.1%)	0.51 (0.29–0.91)	**0.02**
	Frequently	15 (9.9%)	8 (5.3%)	1.60 (0.64–3.99)	0.32
Pre-pregnancy BMI^e^	Normal	99 (71.9%)	119 (78.8%)	1.00 (referent)	
	Underweight	32(21.1%)	16(10.6%)	2.58 (1.31–5.09)	**0.01**
	Overweight	17(11.2%)	15(9.9%)	1.18 (0.55–2.5)	0.68
	Obese	4(2.6%)	1(0.7%)	3.21 (0.34–30.60)	0.31
Educational level	Junior High	36(23.7%)	26(17.2%)	1.00 (referent)	
	Senior High	58(38.2%)	38(25.2%)	1.13 (0.59–2.18)	0.71
	Undergraduate	38(25.0%)	47(31.1%)	0.58 (0.29–1.16)	0.13
	Postgraduate	20(13.2%)	40(26.5%)	0.36 (0.17–0.79)	**0.01**
Family income per month^f^	<1000	22 (14.5%)	6 (4.0%)	1.00 (referent)	
	1000–3000	43 (28.3%)	38 (25.2%)	0.336 (0.12–0.93)	**0.04**
	3001–5000	46 (30.3%)	43(28.5%)	0.32 (0.11–0.88)	**0.03**
	5001–10000	33 (21.7%)	59 (39.1%)	0.19 (0.07–0.55)	**0.002**

^*^Adjusted for maternal age at delivery and residence during pregnancy.

^a^Irregular menstruation during 12 months before pregnancy.

^b^Drug intake during 3 months before pregnancy.

^c^DP: during pregnancy.

^d^Occupational passive smoking exposure ≥15 mins/day: Seldom: 1–2 days per week; Frequently: ≥3 days per week.

^e^Underweight: <18.5 kg/m^2^; Normal: 18.5–24.9 kg/m^2^; Overweight: 25–29.9 kg/m^2^; Obese: ≥30 kg/m^2^.

^f^Unit of family income per month: RMB.

Thereafter, we performed multivariable logistic regression analyses based on these variables (Table 3). The results indicated that the LBW of infants could increase the risk of HS (OR = 4.14, 95%CI: 1.58–10.87, *P* = 0.004). Maternal factors including irregular menstruation (OR = 3.31), passive smoking (seldom: OR = 4.02; frequently: OR = 8.75), underweight BMI (OR = 2.72) before conception, and occurrence of abnormalities (OR = 2.17) during pregnancy significantly increased the risk of HS. However, we also found that compared with no exposure to passive smoking, seldom exposure to passive smoking during pregnancy was associated with reduced risk of HS and the OR was 0.20 (95%CI: 0.06–0.66). The results also showed that higher education level was a protective factor for HS (fathers with undergraduate level: OR = 0.18, 95%CI: 0.04–0.75, *P* = 0.02), and lower education level was a risk factor (mothers with senior high level: OR = 3.90, 95%CI: 1.22–12.45, *P* = 0.02). The decreased risk of HS was also associated with household monthly income (1000–3000: OR = 0.22, 95%CI: 0.07–0.71; 3001–5000: OR = 0.27, 95%CI: 0.08–0.87; 5001–10000: OR = 0.15, 95%CI: 0.04–0.52).

**Table 3:** Multivariable logistic regression analyses of the risk factors for HS.

Risk factors		β	S	Wald	OR(95%CI)	P*
**Of Infant**						
Low birth weight	No				1.00 (referent)	
	Yes	1.42	0.49	8.34	**4.14 (1.58–10.87)**	**0.004**
**Of Father**						
Educational level	Junior High				1.00 (referent)	
	Senior High	−0.96	0.61	2.50	0.38 (0.12–1.26)	0.11
	Undergraduate	−1.72	0.73	5.53	**0.18 (0.04–0.75)**	**0.02**
	Postgraduate	−0.96	0.81	1.40	0.38 (0.08–1.88)	0.24
**Of Mother**						
Irregular menstruation BP^a^	No				1.00 (referent)	
	Yes	1.20	0.43	7.73	**3.31 (1.42–7.71)**	**0.005**
Occurrence of abnormalities DP^b^	No				1.00 (referent)	
	Yes	0.77	0.39	3.85	**2.17 (1.00–4.70)**	**0.05**
Passive smoking exposure BP^c^	No				1.00 (referent)	
	Seldom	1.39	0.58	5.72	**4.02 (1.29–12.58)**	**0.02**
	Frequently	2.17	0.90	5.82	**8.75 (1.50–50.93)**	**0.02**
Passive smoking exposure DP^c^	No				1.00 (referent)	
	Seldom	−1.60	0.61	6.97	**0.20 (0.06–0.66)**	**0.01**
	Frequently	−1.10	0.98	1.26	0.33 (0.05–2.27)	0.21
Educational level	Junior High				1.00 (referent)	
	Senior High	1.36	0.59	5.26	**3.90 (1.22–12.45)**	**0.02**
	Undergraduate	1.07	0.71	2.25	2.90 (0.72–1.67)	0.13
	Postgraduate	0.54	0.79	0.47	1.71 (0.37–8.03)	0.50
Pre-pregnancy BMI^d^	Normal				1.00 (referent)	
	Underweight	1.00	0.43	5.36	**2.72 (1.17–6.36)**	**0.02**
	Overweight	−0.10	0.46	0.05	0.90 (0.37–2.22)	0.82
	Obese	0.77	1.40	0.30	2.16 (0.14–33.79)	0.58
Family income per month^e^	<1000				1.00 (referent)	
	1000–3000	−1.51	0.60	6.38	**0.22 (0.07–0.71)**	**0.01**
	3001–5000	−1.31	0.59	4.86	**0.27 (0.08–0.87)**	**0.03**
	5001–10000	−1.91	0.64	8.84	**0.15 (0.04–0.52)**	**0.003**
	>10000	−0.90	0.90	1.02	0.41 (0.07–2.35)	0.31

*Adjusted for maternal age at delivery, and residence during pregnancy.

^a^Irregular menstruation during 12 months before pregnancy.

^b^During pregnancy.

^c^Occupational passive smoking exposure ≥15 mins/day: Seldom: 1~2 days per week; Frequently: ≥3 days per week.

^d^Underweight: <18.5 kg/m^2^; Normal: 18.5–24.9 kg/m^2^; Overweight: 25–29.9 kg/m^2^; Obese: ≥30 kg/m^2^.

^e^Unit of family income per month: RMB.

### Association between *CYP1A1/CYP17A1* genotypes and hypospadias risk

Genotype frequencies of *CYP1A1* rs1048943 and *CYP17A1* rs4919686 polymorphisms were in HWE (*P* > 0.05) and the distributions of genotypes and mutant alleles were shown in Table 4. The distribution of TT, TC and CC genotypes of *CYP1A1* rs1048943 was 52.2%, 39.2% and 8.6% respectively, and the distribution of AA, AC and CC genotypes of *CYP17A1* rs4919686 was 58.8%, 33.1% and 8.1% respectively. After adjusted for confounding factors, we found that the homozygous mutant genotype CC of *CYP1A1* rs1048943 significantly increased the risk of HS (OR = 4.87, 95%CI: 1.25–18.94, *P* = 0.022). In addition, homozygous mutant genotype CC and recessive genotype AC + CC of *CYP17A1* rs4919686 revealed a significantly higher risk of HS in contrast to the AA genotype (CC vs AA: OR = 5.82, 95% CI: 1.45–23.4, *P* = 0.013; AC + CC vs AA: OR = 2.17, 95% CI: 1.24–3.80, *P* = 0.007). Furthermore, the mutant allele C of *CYP17A1* rs4919686 also showed an increased risk of HS (OR = 1.77, 95%CI: 1.17–2.66, *P* = 0.006).

**Table 4:** Effect of CYP1A1 rs1048943 and CYP17A1 rs4919686 polymorphisms on the risk of HS.

Gene	Case (n = 148) No. (%)	Control (n = 147) No. (%)	Crude OR	Adjusted OR	95%CI	P*	*P* for HWE
***CYP1A1*** **rs1048943**							0.14
TT	77 (52.2%)	81 (55.1%)		Ref (1.00)			
TC	58 (39.2%)	62 (42.2%)	0.94	0.90	0.52–1.57	0.72	
CC	13 (8.6%)	4 (2.7%)	4.44	**4.87**	**1.25–18.94**	**0.02**	
TC + CC	71 (47.8%)	66 (44.9%)	0.91	0.94	0.55–1.61	0.82	
T	212 (71.6%)	224 (76.2%)		Ref (1.00)			
C	84 (28.4%)	70 (23.8%)		1.27	0.88–1.83	0.21	
***CYP17A1*** **rs4919686**							0.93
AA	87 (58.8%)	104 (70.7%)		Ref (1.00)			
AC	49 (33.1%)	40 (27.2%)	1.406	1.80	1.01–3.20	1.80	
CC	12 (8.1%)	3 (2.1%)	4.838	**5.82**	**1.45–23.4**	**0.01**	
AC + CC	61 (41.2%)	43 (29.3%)	1.64	**2.17**	**1.24–3.80**	**0.01**	
A	223 (75.3%)	248 (84.4%)		Ref (1.00)			
C	73 (24.7%)	46 (15.6%)		**1.77**	**1.17–2.66**	**0.01**	

*Adjusted for maternal age at delivery, and residence during pregnancy.

### Interactions between risk factors and *CYP1A1/CYP17A1* polymorphisms

2 × 4 cross-tabs was adopted to detect the gene-environment interactions (Supplementary Table 1), meanwhile, “RR_11_ − RR_00_ > (RR_10_ − RR_00_) + (RR_01_ − RR_00_)” was defined as additive interaction of gene-environment. Although both of the results of gene-environment interaction were not indicated any significant difference between the groups of case and control, the additive gene-environment interactions were found in several models (Table 5), including interactions of *CYP1A1* with irregular menstruation before conception, passive smoking before conception and occurrence of abnormalities during pregnancy, as well as interactions of *CYP17A1* with occurrence of abnormalities during pregnancy.

**Table 5:** Effect of interaction of CYP1A1/CYP17A1 polymorphisms-environmental risk factors on the risk of HS.

Gene	Risk factors	Cases	Controls	OR (95%CI)^*^	RERI (95%CI)^*^	AP (95%CI)^*^
**Of Mothers**						
***CYP1A1*** **rs1048943**	Irregular menstruation BP^a^					
TT	No	58	57	Ref (1.00)	4.27 (−2.49–11.03)	0.72 (0.26–1.17)
TC + CC	No	55	77	0.71 (0.40–1.23)		
TT	Yes	13	8	1.98 (0.06–5.98)		
TC + CC	Yes	22	5	5.96 (1.89–18.85)		
***CYP1A1***	Occurrence of abnormalities DP^b^					
TT	No	39	47	Ref (1.00)	0.31 (−2.23–2.85)	0.12 (−0.80–1.04)
TC + CC	No	54	54	0.98 (0.53–1.80)		
TT	Yes	32	18	2.28 (1.04–4.98)		
TC + CC	Yes	23	10	2.57 (1.00–6.55)		
***CYP1A1***	Passive smoking BP					
TT	No	45	42	Ref (1.00)	0.11 (−1.10–1.32)	0.11 (−1.01–1.23)
TC + CC	No	48	53	0.83 (0.44–1.57)		
TT	Yes	26	23	1.10 (0.50–2.41)		
TC + CC	Yes	29	29	1.04 (0.41–2.66)		
***CYP17A1***	Occurrence of abnormalities DP					
AA	No	57	81	Ref (1.00)	6.98 (−3.39–17.35)	0.77 (0.48–1.06)
AC + CC	No	36	39	1.30 (0.69–2.47)		
AA	Yes	31	23	1.74 (0.86–3.52)		
AC + CC	Yes	24	4	9.02 (2.78–29.22)		

^*^Adjusted for maternal age at delivery, educational level and residence during pregnancy.

^a^Before pregnancy.

^b^During pregnancy.

## Discussion

As we have known, most chronic and complex diseases are likely caused by the interactions among environmental exposure, genetic polymorphisms, and lifestyle behaviors. HS remains to be a prevalent congenital malformation in male reproductive system, with a multifarious etiology and challenging operation plans^37^. A total of 152 live born children diagnosed with HS, 151 cases of controls without HS in hospital during the same period, and their parents were included in this study to investigate the risk factors for HS, the roles of *CYP1A1/CYP17A1* polymorphisms, and the gene-environment interaction in the etiology of HS in Chinese population.

We found several factors based on questionnaire data that were associated with HS risk including LBW of infant, parental educational level, maternal factors such as passive smoking before and during pregnancy, irregular menstruation before pregnancy, occurrence of abnormalities during pregnancy and preconception BMI, and household monthly income.

LBW of infant has already been confirmed as a risk factor for hypospadias in many studies^38–42^. In this study, we found that infants with LBW had a more than 4-fold increased risk for HS. A study conducted in Western Siberia by Osadchuk *et al*. recently revealed that frequent drinking and smoking in young men could alter the hormonal and metabolic balance and would increase the risk of reproductive disorders ultimately^43^. However, Carmichael *et al*. found that pregnant women exposed to secondhand smoke at home in early pregnancy was associated with reduced HS risk, which was similar with our investigation about seldom maternal exposure to secondhand smoke during pregnancy (OR = 0.20)^44^. Nevertheless, for exposure to passive smoking before conception in present study, significant increased trends for HS risk were observed in seldom and frequently exposure compared to non-exposure, which might due to constitution of mothers before conception was more sensitivity and vulnerable. Irregular menstruation has been considered as a women’s poor reproductive health and a marker of metabolic disorders^45^, therefore, it might disrupt the balance of hormones *in vivo*. Our finding indicated that irregular menstruation before conception was a risk factor as well. Occurrence of abnormalities during pregnancy in this study, including gestational diabetes, amniotic fluid anomaly, toxaemias of pregnancy, influenza and premature delivery, was found to increase the risk, which was similar with the results of published papers^38,46,47^. Akre *et al*. found that infants born to obese mothers (BMI: ≥30 kg/m^2^) was associated with a more than 2-fold increased risk of HS compared with infants born to mothers with a normal weight (BMI: 20–24 kg/m^2^)^48^. However, Adams *et al*. found that maternal obesity was not a cause of HS in male infants^49^. Our results showed that HS risk was neither associated with overweight (BMI: 25–29.9 kg/m^2^) nor with obese (BMI: ≥30 kg/m^2^), but with underweight (BMI: <18.5 kg/m^2^) with an OR of 2.72, which was consistent with the results of Rankin *et al*.^50^. The higher education level was a protective factor for HS and the lower education level was a risk factor, which might on account of the fact that parents with a high educational level would have a better job and got well-paid so that pregnant women could get better care. Meanwhile, our study also indicated that household monthly income >1000 RMB significantly reduced the risk of HS, which might due to that for women from wealthy families known how to fend for themselves as well as have a comprehensive nutritional meal.

The biosynthesis and bioactivation of endogenous sex hormones, including estrogen and androgen, are necessary for the normal development of genital gland, which are catalyzed by enzymes of CYP1A1 and CYP17A1^51^. As far as we know, the association between maternal *CYP1A1/CYP17A1* SNPs and hypospadias have not been reported in Chinese population, let alone gene-environment interaction. In this study, analysis of the distributions of SNPs in *CYP1A1* gene showed that mothers with CC mutant genotype in *CYP1A1* increased the risk of HS more than 4-fold. On the contrary, a study conducted in Japan population (including 31 case mothers and 64 control mothers) by Kurahashi *et al*. showed that mutant genotypes (TC and TC + CC) in *CYP1A1* were associated with decreased risk of HS^52^. The different results might be attributed to the existence of population differences and the different sample size, so further study with larger sample size and different populations is needed to confirm this association. In addition, the *CYP17A1* rs4919686 polymorphism was also significantly associated at both allelic and genotypic levels with risk of HS (CC: OR = 5.82; AC + CC: OR = 2.17; allele C: OR = 1.77), which is similar with the results reported by Qin *et al*.^53^. Hence, SNPs of rs1048943 in *CYP1A1* and rs4919686 in *CYP17A1*, which are involved in the bio-synthesis of sex hormones, have major roles in normal development of male genitalia, and may result in HS by changing the activity of the corresponding enzymes.

The pathogenesis of hypospadias is viewed as multifactorial, and induced by genes polymorphisms and gene-environment interactions, hence, it is necessary to investigate the roles of interaction between *CYP1A1/CYP17A1* polymorphisms and environmental risk factors. In this study, the positive additive interactions were observed in several models (Table 5). Evidence showed that mothers with recessive genotypes (TC + CC) of *CYP1A1* could interact with risk factors, including irregular menstruation, passive smoking before conception and occurrence of abnormalities during pregnancy, which resulted in the increased HS risk in male offsprings. The interaction between passive smoking with *CYP1A1* rs1048943 polymorphism was also associated with other diseases, such as coronary artery disease^54^ and hypertension^55^. It is noteworthy that both of the two SNPs in *CYP1A1* and *CYP17A1* had additive interactions with occurrence of abnormalities during pregnancy, which indicated that occurrence of abnormalities was a definite risk factor for HS.

Here, this is by far the first comprehensive report in regard to the interactions of *CYP1A1/CYP17A1* polymorphisms with environmental risk factors before and during pregnancy for the risk of HS in a Chinese population. Despite all this, our study still have several limitations. First, the nature of our study is case-control study and data was collected by interviewing the parents of cases and controls retrospectively. Hence, the validity of these data could not be verified and inaccurate data might lead to misclassification due to recall bias. In order to reduce uncertain information, questions of most factors were relatively easy to remember, the interviewer was well-trained, and all statistical analysis were adjusted for confounding factors, so the results are credible. Second, the study group was relatively small and the number of studied SNPs was limited. In addition, the association of HS phenotypes with gene polymorphisms was not investigated and our participants only include Chinese population. Therefore, an upgraded version of our investigation including larger sample size with more SNPs of different ethnics groups is required to confirm our findings, what’s more, the interaction of different phenotypes with risk factors are also needed to explore.

## Conclusions

Overall, this study suggests that maternal *CYP1A1/CYP17A1* polymorphisms involved in metabolism and synthesis of endogenous hormones might have a significant role in the risk of development of HS in the offsprings. In addition, several environmental risk factors are associated with HS risk and can interact with *CYP1A1/CYP17A1* polymorphisms in Chinese population. Using gene-environment interaction model can elucidate the etiology of HS clearly, and provide more efficient measures. Although our study as yet still have several limitations, we stress the importance of performing stratified analyses for the different phenotypes of HS in further research.

## Supplementary information

Supplementary Table 1 2×4 cross-tab on gene-environment interaction

## Data Availability

The data that support the findings of this study are available from [Shengjing Hospital of China Medical University and Shenyang Medical College] but restrictions apply to the availability of these data, which were used under license for the current study, and so are not publicly available. Data are however available from the authors upon reasonable request and with permission of [Shengjing Hospital of China Medical University and Shenyang Medical College].
