# Supplementary material for: Interaction between CYP1A1/CYP17A1 polymorphisms and parental risk factors in the risk of hypospadias in a Chinese population
Source: Sci Rep. 2019 Mar 11;9:4123. doi: 10.1038/s41598-019-40755-8 (PMC6411735; doi:10.1038/s41598-019-40755-8)
Supplement: Supplementary file 1 — Supplementary Table 1 2×4 cross-tab on gene-environment interaction [file 41598_2019_40755_MOESM1_ESM.doc]

**Title Page**

**Interaction between CYP1A1/CYP17A1 polymorphisms and parental risk factors in the risk of hypospadias in a Chinese population**

**Running title:** Interaction of CYP1A1/CYP17A1 polymorphisms-environmental risk factors & hypospadias

**Yaping Mao^1,2#^, Kang Zhang^1#^, Lin Ma^1^, Xiaoyun Yun^3^, Fengrong Ou^3^, Ge Liu^4^, Yi Yang^4^, Yumin Zhang^1^, Xiucong Pei^1^, Zhiwen Duan^1^, Mingyue Ma^1*^**

^1^Department of Toxicology, School of Public Heath, Shenyang Medical College, Shenyang, Liaoning Province 110034, China

^2^Editorial Department of Journal of Shenyang Medical College, Shenyang Medical College, Shenyang, Liaoning Province 110034, China

^3^Department of Clinical Nutrition, The First Affiliated Hospital of China Medical University, Shenyang, Liaoning Province 110001, China

^4^Department of Pediatric Surgery, Shengjing Hospital of China Medical University, Shenyang, Liaoning Province 110004, China

^#^ Co-first author: The two authors contributed equally to this work

^*^ Corresponding author: Mingyue Ma

Email: [mymacmu@163.com](mailto:mymacmu@163.com)); mamingyue@symc.edu.cn

Tel: +86-24-62253260

Fax: +86-24-62215829

Supplementary Table 1 2×4 cross-tab on gene-environment interaction

| Gene | Environmental Factor | No. Cases | No. Controls | OR | Information |
| --- | --- | --- | --- | --- | --- |
| -^a^ | -^a^ | a | b | 1 (OR_00_) | Reference |
| +^b^ | - | c | d | OR_10_ | G^*^ |
| - | +^b^ | e | f | OR_01_ | E^**^ |
| + | + | g | h | OR_11_ | G×E^***^ |

^a^ Gene : wild genotype

Environmental factors: no risky exposure

^b^ Gene: variant genotype ( heterozygote and homozygote)

Environmental factors: risky exposure

^*^ Mutant genotype effect

^**^ Environmental risk factors exposure effect

^***^ Effect of gene-environment factors co-exist
